# The development of a technologic approach to improve access to individualized clinical documentation in caregivers’ preferred language

**DOI:** 10.1371/journal.pdig.0001721

**Published:** 2026-09-11

**Authors:** Jacqueline Toscano, King Yan Kwok, Meg Simione

**Affiliations:** 1 Department of Communication Sciences and Disorders, MGH Institute of Health Professions, Boston, Massachusetts, United States of America; 2 Boston Public Schools, Roxbury, Massachusetts, United States of America; 3 Department of Communicative Disorders, College of Health Sciences, University of Rhode Island, Kingston, Rhode Island, United States of America; Khomein University of Medical Sciences, IRAN, ISLAMIC REPUBLIC OF

## Abstract

This pilot study aims to describe the process of developing a template that semi-automatically translates information from English to Spanish to improve caregivers’ access to clinical documents in their preferred language. It used human-centered design and the Discover, Design/Build, and Test Framework to improve health literacy outcomes for patients who use a language other than English. The Discover Phase revealed current methods and barriers to clinicians providing patients access to written documentation in their primary language. During the Design/Build Phase, an interprofessional team of a speech-language pathologist and a certified translation specialist developed the template in the electronic health record. In the Test Phase, we evaluated the template’s acceptability and feasibility and surveyed speech-language pathologists (SLPs) and patients’ caregivers. Clinicians affirmed the importance of the template, but also had concerns regarding feasibility and usability. Caregivers found it helpful to receive their child’s health information in their primary language. The results showed that sustainable access to written documentation in patients’ preferred language is lacking, and this template is prepared to reduce language barriers in an overwhelmed healthcare system.

## Author summary

In this pilot study, we describe how we created a template that semi-automatically translates pediatric speech-language evaluation results from English into Spanish, without requiring the user to know Spanish. We used the Discover, Design/Build, and Test Framework to evaluate the template’s usability among clinicians and patient caregivers. In the Discover phase, we identified current methods and barriers to health literacy when English-speaking clinicians communicate evaluation results to their pediatric patients’ caregivers. In the Design/Build Phase, a speech-language pathologist and certified translation specialist developed the template. In the Test Phase, we evaluated the template’s usability using quantitative and qualitative measures. The results showed that clinicians found the template valuable and suggested ways to improve its usability. Caregivers were enthusiastic about receiving their child’s health information in their primary language. The results revealed limited access to written documentation in patients’ preferred language. Therefore, this template may reduce language barriers in an overwhelmed healthcare system.

## Introduction

In the United States, more than 20% of households speak a language other than English (LOE), making health literacy in an English-dominant healthcare system difficult to achieve [1]. There are considerable barriers to accessing and understanding written health information, ultimately leading to poor health outcomes, caused by issues such as confusion about treatment strategies and early detection guidelines due to lack of understanding, for those whose dominant language is not English [2–4]. The United States National Institutes of Health (NIH) set health literacy goals that highlighted a need for reliable methods of translating written patient information [5]. The best methods to improve health literacy for patients who use a LOE for clinical documentation require technological advancements and human collaboration, which is rarely done.

It is documented that the cost, time, and workload of certified translation specialists can be insurmountable when considering the needs of a hospital system [6], leading to reduced usability in clinical settings [7]. While machine translation (e.g., BabelDr, Medibabble, UniversalDoctor) [8] may seem like a reasonable solution and has proven to bridge language barriers, it is not yet prepared for confidential and context-dependent patient information [9] and must still be monitored by human translators [10]. The current literature indicates a lack of standardized best practices for translating individualized clinical documentation [5]. Few strategies exist that provide individualized clinical documentation in a patient’s (or caregiver’s) primary language, increase access to their health information in a timely yet accurate manner, and meet the needs of clinicians and healthcare organizations. Human-centered design is a technology development approach rooted in user experience that was used to develop and assess the usability of new methods to improve health literacy for patients who use an LOE, allowing for stakeholder input and the prioritization of their needs [7,11]. The purpose of this pilot study is to describe the process of using human-centered design to develop a template for pediatric speech and language evaluations that semi-automatically populates translated information from English to Spanish to improve health literacy for patients who use an LOE.

## Methods

### Study overview

In this pilot study, we used human-centered design guided by the Discover, Design/Build, and Test (DDBT) framework [11]. The DDBT framework consists of three phases to identify and overcome challenges through stakeholder input and their experience with the innovation with a goal of positively impacting the innovation’s usability.

We conducted the study with pediatric speech-language pathologists (SLPs) and caregivers of children at an academic medical center in Boston, MA, with community health centers in neighboring cities. The sites selected have a higher percentage of non-English speaking households than the national average [12]. As part of their role, SLPs conduct speech and language evaluations and then provide written reports with care recommendations to caregivers. In accordance with the standard of care, monolingual clinicians are expected to complete bilingual evaluations using interpreter services. The study was approved by the Mass General Brigham (MGB) Institutional Review Boards.

### Discover phase

The first phase of the DBBT framework is to identify stakeholders and challenges that need to be addressed. The Discover phase included informal discussions with clinicians about their current report templates and how they could be developed into a translated version. Additionally, we reviewed institutional translation policies and practices using the hospital’s online policy portal and informal discussions with administrative personnel. Specifically, the policies we reviewed pertained to which staff can participate in written translations, as well as the cost and time needed for such work. We also investigated how SLPs work with interpreter services to communicate with the diverse population of patients seeking their services and collected and reviewed templates currently being used.

### Design/build phase

In Phase Two, a bilingual Spanish-English SLP (JT) and a certified translation specialist (or Certified Healthcare Interpreter; CHI) developed the template through a series of synchronous consultations and several email exchanges over a period of two months. The template aimed to bridge gaps created by the current unsustainable methods of high-demand written translation. Using information gathered in Phase One, the team created a pilot template for the translated evaluation letter (i.e., letter written for caregivers with an abbreviated version of the evaluation results and recommendations translated into their preferred language) in English and Spanish. The SLP developed the template in English and then generated an initial translation. The CHI revised and, as appropriate, wrote formal Spanish iterations of the template while maintaining constant collaboration with the bilingual Spanish-English SLP via email. Over about two months, the CHI and SLP had two virtual meetings and ongoing email correspondence, in which they discussed semantic, syntactic, and social-pragmatic considerations for the translation regarding the accuracy of content, especially considering the clinical context.

### Test phase

In Phase Three, we recruited nine monolingual and bilingual clinicians via email for the study. Clinicians completed surveys at two different time points in Phase Three. They completed the first survey prior to using the template, and then a subset of clinicians completed the second survey following the use of the template. Most clinicians who participated in the first survey did not opt in to participate in the second survey, primarily due to projections that they might not evaluate Spanish-speaking patients within the timeframe of this study. The first survey elicited information about their current practices in communicating speech and language evaluation results and recommendations to non-English-speaking caregivers, the current need for a template that translates this content into Spanish, and willingness to recruit caregivers with whom to trial the template. The first survey was adapted from surveys used to evaluate intervention approaches [13,14].

A subset of three clinicians completed the second survey about their experience using the template, as well as its acceptability, appropriateness, and feasibility, adapted from the Acceptability of Intervention Measure (AIM), Intervention Appropriateness Measure (IAM), and Feasibility of Intervention Measure (FIM) [15]. Both surveys consisted of multiple-choice and open-response items about the template and were completed via a web-based survey program (REDCap).

Caregivers were recruited during or after their initial evaluation visit. Inclusion criteria included native and/or dominant use of the Spanish language and status as a caregiver of a patient aged 2–5 years. We initially identified nine caregivers to participate in this pilot. Three did not respond or declined to complete the survey post-recruitment, two opted for email surveys but did not complete them, and one missed the timeframe of the data collection period. Therefore, the test phase included three caregiver participants. Caregivers received the completed template (i.e., a letter with translated evaluation results). Then they completed an adapted version of a survey used to investigate the impact of health interventions [16]. The surveys included multiple-choice and open-response items. Caregivers could complete the survey on the web or by phone.

### Quantitative and qualitative data analyses

Clinician input from the Discover phase was informally analyzed and incorporated into subsequent phases. Descriptive statistics, including counts and frequencies, were calculated for clinician and caregiver multiple-choice survey questions. Microsoft Excel software (version 16.74) was used for statistical analyses.

Open-response survey items elicited qualitative feedback, and framework-guided rapid analysis methods were used [17]. Two primary analysts and a reviewer made up the analysis team. Analysts completed summary tables using Microsoft Word for each survey, and the reviewer provided feedback. The primary analyst then used the data in the summary tables to identify and arrange themes, subthemes, and exemplar quotes into three matrices (i.e., one for each survey) using Microsoft Excel. The analysis team reviewed the matrices to finalize themes and selection of exemplar quotes.

## Results

### Discover phase

After speaking with clinicians about their current practice and reviewing policies, we found that policy boundaries protected interpreters’ time by prohibiting them from translating full-length written documents, such as evaluation reports, into other languages. The permitted option was to submit the written document for formal translation through a translation services department. However, this service was rarely used, given its high costs and long wait times, especially with high demand. With multiple clinicians writing evaluation reports at least weekly, the financial cost and time delay were not sustainable. LOE families were often sent home with reports in English that they could not read. During this phase, we also learned about templates clinicians had developed in English to make their report writing more efficient. These templates included test descriptions, terms and phrases commonly used for assessment analysis, and tables for reporting assessment results.

### Design/build phase

During this phase, we designed a template built into the electronic medical record (EMR) using Epic SmartPhrase technology. SmartPhrase templates include functions that link data to the document, offer drop-down menus for single- or multi-select options, and allow for editing capabilities to individualize documentation to the patient and circumstance. To ensure usability, the template used a series of drop-down menus and comment banks that included both the English and Spanish versions of each option (see S1 File). The template’s comment banks require clinicians to copy and paste, from an option bank, impressions that apply to their patients. After completing all the drop-down menus and comment banks and deleting the English guides, clinicians are left with an abbreviated translation of their evaluation findings and recommendations in Spanish.

### Test phase

#### First clinician survey.

Of the SLPs who completed the first survey (n = 9), all spoke English as their primary language, and six of the nine clinicians practiced between 11 and 20 years (Table 1). They all reported evaluating patients whose caregivers primarily speak Spanish, with most (6 of 9) using interpreter services for communication with these caregivers. Clinicians who completed the first survey reported a strong need for the template (8 of 9) and a desire to use it in their practice (8 of 9). They responded that the template would be completely (5 of 9) or somewhat (4 of 9) useful in their clinical practice, including allowing for “more transparency of information from the initial evaluation in written format” (Table 2).

**Table 1:** Quantitative results of first clinician survey (n = 9).

Item	n/N
Years practicing as a certified speech-language pathologist (SLP)	
1-5 years	3/9
11-20 years	6/9
Primary language is English	9/9
Evaluates patients whose caregivers primarily speak Spanish	9/9
Current method of communicating results*	
Provide oral summary during appointment or call	7/9
Orally translate results during appointment or call	3/9
Other (Uses interpreter services, provides less detail verbally)	2/9
Current method of communicating recommendations*	
Provide an oral summary during appointment or call	6/9
Orally translates recommendations during appointment or call	3/9
Uses interpreter services	2/9
Usefulness in clinical practice	
Completely useful	5/9
Somewhat useful	4/9
Need for the letter	
Strong need	8/9
Some need	1/9
Degree to wanting to use the letter	
Definitely want to use	8/9
Somewhat want to use	1/9

*Note. Clinician respondents could select multiple responses and enter a free text response for this survey item.

**Table 2:** Qualitative results of first clinician survey (n = 9).

Themes	Exemplar Quotes
**Current Practices Communicating Evaluation Outcomes**	
Clinicians work with an interpreter to communicate evaluation results.	“I use a qualified bilingual interpreter to… provide an oral summary of evaluation highlights.”
**Benefits to the Letter**	
The letter would have an impact on accessibility of patient health information.	“It contributes to the patient experience, and I imagine helps them feel their language and culture is valued.”
The letter would promote increased caregiver understanding of findings/recommendations.	“It allows for more transparency of information from the initial evaluation in written format…”
**Drawbacks to the Letter**	
No suspected drawbacks.	“I don’t see any drawbacks.”
The letter will generate additional work and require additional time for the clinician.	“… It is a separate step to complete within the evaluation documentation process, but it is helpful and worth it for families.”
Potential for misunderstanding and/or missing information.	“Potential for information to be left out that is in the original evaluation.”

### Second clinician survey

After using the template, three (n = 3) clinicians completed the second survey (see Table 3). The SLPs reported they found the template somewhat acceptable and appropriate (2 of 3) or very acceptable and appropriate (1 of 3). The qualitative responses also affirmed the need for the template (Table 4). For example, one clinician wrote, “I think/hope that patients will feel more like they are perceived as a valued member of the team to help their child and that the letter will give them agency in taking next steps.” All SLPs found the template to be somewhat feasible, but had concerns about the ease of use. One SLP responded, “It took a long time to complete.” Suggested adaptations included additional detail in certain sections and the need for a more efficient workflow.

**Table 3:** Quantitative results of second clinician survey (n = 3).

Item	N/N
Qualified Bilingual Staff member in Spanish	2/3
Uses interpreter services to review evaluations with caregivers	3/3
Languages for which interpreter services are solicited*	
Portuguese	3/3
Arabic	2/3
Russian	1/3
Spanish	1/3
Mandarin	1/3
Other	1/3
*Acceptability*	
Clinician approval of letter	
Somewhat meets their approval	2/3
Does not meet their approval	1/3
Letter appeal to clinician	
Completely appealing	1/3
Somewhat appealing	2/3
*Intervention Appropriateness*	
Letter applicability	
Completely applicable	2/3
Somewhat applicable	1/3
Match of letter to clinician need	
Completely good match	1/3
Somewhat of a good match	1/3
Not a good match at all	1/3
*Feasibility*	
Implementability of the letter	
Somewhat implementable	3/3
Ease of letter	
Somewhat easy to use	2/3
Not easy to use at all	1/3

*Note. Clinician respondents could select multiple responses for this survey item.

**Table 4:** Qualitative results of second clinician survey (n = 3).

Themes	Exemplar Quotes
**Acceptability**	
The clinician(s) liked being able to increase their patients’ caregivers’ access to clinical information.	“Being able to provide summary and recommendations in patient’s native language!!”
The clinician(s) disliked that the letter took a long time to complete.	“It took a long time to complete - 2+ hours - for monolingual English speaker first time.”
**Feasibility**	
The Epic SmartPhrase software features are helpful.	“The "multiple choice" nature of the report was also helpful.”
There is a lack of cohesiveness in the template.	“There was a lot of deleting and rearranging that needed to be done to make the letter seem cohesive.”
The process of completing the letter was cumbersome.	“… it felt like I was doubling my work sometimes or saying things not exactly as they were in my English report…”
The clinician(s) questioned the likelihood that the caregiver will review the translated report.	“I wonder how much patients look at the written report even in their language”
**Benefits to Template Identified After Using The Letter**	
The patient may perceive themselves as a valued member of team.	“I think/hope that patients will feel more like they are perceived as a valued member of the team to help their child and that the letter will give them agency in taking next steps.”
Patients and families will experience increased access to health and educational information in their home language.	“It’s a great step to have written information in a patient’s language and consistent with accessing health and educational information in a home language.”
**Suggested Adaptations to The Template**	
There are suggested adaptations for the translation template as a whole.	“…if there is more specific information for certain areas, can there be a way to reflect that (i.e. select statement re: more specific information gathered, please refer to clinician).”

### Caregivers’ survey

All caregivers (n = 3) reported their dominant/preferred language as Spanish (see A.1). After receiving the letter, all caregivers reported that they found it to be very helpful and very easy to understand (Table 5). All were very satisfied with the ability of the letter to explain evaluation results, but were mixed regarding the comprehensibility of the results. Caregivers indicated a positive impact of receiving the letter and wanted the letter to be used by other departments. One caregiver responded, “We are going to be more sure and conscious of what is happening with our child.” (Table 6).

**Table 5:** Quantitative results of caregiver survey (n = 3).

Item	n/N
Dominant and preferred language is Spanish	3/3
Age of child being evaluated	
Between 2–3 years old	1/3
Between 3–5 years old	2/3
Received evaluation letter in Spanish	3/3
Impression of evaluation results in Spanish	
Very helpful	3/3
Very easy to understand	3/3
Not difficult at all to understand	2/3
Very difficult to understand in Spanish	1/3
Likelihood of recommending this letter to another family	
Very likely	2/3
Degree of benefit of receiving these results	
Significant benefit	2/3
Somewhat of a benefit	1/3
Degree of satisfaction with letter’s ability to explain outcomes	
Very satisfied with ability of the letter to explain results	3/3
Very satisfied with letter’s ability to explain recommendations	2/3
Somewhat satisfied with letter’s ability to explain recommendations	1/3

**Table 6:** Qualitative results of caregiver survey (n = 3).

Themes	Exemplar Quotes
**Positive Impact**	
The letter had positive impacts on the caregiver’s experience.	“…we are going to be more sure and conscious of what is happening with our child.”
**Negative Impact**	
The letter did not have any negative impacts on the caregiver’s experience.	“No -well- I believe there wouldn’t be any negative impacts”
**Suggested Adaptations to The Letter**	
There are not any suggested adaptations for the letter.	“No, for me it was perfect. Let’s see, how can the letter be improved? No, because I saw that it came out super well. It arrived on time, super specific. No, I don’t see an aspect to improve.”
**Follow-up Questions about The Letter**	
There are not any follow-up questions about the letter itself.	“… Is it going to be only with -with with- [sic] the language therapies, or is it going to be now -like- at the full level of [child’s name]’s health, in other words, all the other departments.”

### Implementing adaptations

Based on the feedback from clinicians and caregivers, we can now adapt the template and continue to use DBBT cycles of feedback and revision. Adaptations will include streamlining the content to avoid redundancy, adding clinical areas not included in the initial version, and integrating it more seamlessly into the workflow to reduce clinician burden.

## Discussion

In this study, we employed a DBBT framework to develop a translated evaluation template for speech and language evaluations with the goal of improving access to patient information for caregivers who use a LOE. We aimed to understand current practices and stakeholders’ needs, design and build a semi-automatic translation template that meets those needs, and then evaluate the template in clinical practice.

Clinicians found the template necessary to improve their patients’ access and requested reasonable adaptations for increased efficiency. They found the template cumbersome to use, impacting feasibility, which highlights opportunities for revisions. Studies have shown that inefficiencies in documentation and workflow can hinder patient-centered care, underscoring the need for tailored approaches to improve communication and accessibility [18]. Similar results have been found in studies assessing machine translation methods (e.g., Google Translate, ChatGPT) with clinician evaluators noting performance inconsistencies and showing a preference for professional translation in some instances [19]. Despite the technical inconveniences noted by clinicians, they affirmed the strong need for a template to improve patient access to reports. Caregivers were supportive in their feedback about the translations they received. They strongly appreciated receiving their children’s evaluation results in their preferred language. These outcomes are consistent with recent findings that patient experience improves with language concordance and high-quality interpretation [20].

The need for innovative translation practices is well-documented in the literature and is highlighted by the NIH’s health literacy goals for 2030 [6]. This need was reflected in our data by the caregiver feedback on having access to written documentation in their language. Similar to this study’s findings, experts have reasoned that access to accurate translations allows patients to be more confident participants in their healthcare journeys [6]. With health outcomes of patients who use a LOE falling below those of their English-proficient counterparts (e.g., patients who use LOE experiencing longer hospital stays yet more limited use of preventative care), data demonstrate language inequities across the healthcare setting [21]. Therefore, patients and providers need an immediate and reliable solution to break down written language barriers in healthcare.

Tools such as Google Translate have been attempted to improve equitable access, but machine output inconsistencies urge caution and encourage continued need for human monitoring [22]. Experts warn providers to monitor their translation input (e.g., reduce colloquialisms) to avoid the expected (and potentially life-threatening [22]) errors that Google Translate can output. In the category of machine translation [23], clinicians identified almost 30% of Google Translate’s errors as clinically significant, warning providers against solely using machine translation methods. The involvement of human intelligence continues to be best practice to support advancing technology [24]. However, human translation is a much-needed yet hard-to-attain resource due to barriers such as long waits [23] and high costs [25]. In a patient-centered yet overwhelmed healthcare system, providers need sustainable options to provide consistent written translation to their patients. For institutions that must pay and wait for written translations, this template may relieve staff from requesting multiple translations of similar documentation (e.g., speech-language evaluation reports) and meet the needs of caregivers. The current study shows clinicians are motivated to increase patient language accessibility but require a convenient and sustainable method. Based on the results of this study, this template is a step toward increased accessibility within clinically feasible means. Given that this semi-automatic translation template can be easily built within an EMR system, this approach may be able to be applied across departments, healthcare systems, and languages using a similar collaborative approach.

The current study presented limitations that can be addressed in future iterations of this work. Our study was a pilot with a small sample size at one academic medical center and therefore cannot establish population-level acceptability or feasibility. Further investigation of caregiver and clinician perspectives should include a greater sample size to promote generalizable results. This investigation only focused on one type of speech and language evaluation in a pediatric outpatient setting. Future work should occur both within and across SLP teams (e.g., inpatient and outpatient) and across disciplines to adapt the template to each group’s needs. Furthermore, the template should be revised based on clinician feedback to promote the feasibility of its use to improve patient access outcomes.

## Conclusion and practice implications

This study aimed to describe the process of developing a usable translation template for pediatric speech and language evaluations using human-centered design. The study team investigated current translation practices and clinicians’ needs for efficient translation practices and then developed a semi-automatic template that translates an evaluation report into a caregiver’s primary language. Clinicians found the template to have importance for practice and suggested its feasibility for implementation, with suggested revisions to improve its usability. Caregivers were enthusiastic about the template and receiving the speech and language evaluation in their primary language. The template stands to reduce language barriers and improve health literacy outcomes.

## Supporting information

S1 File Translated evaluation template. Sample of template used for translating patient evaluation reports into Spanish from English. Note. In the template, the user was prompted with the following message: “***Note to clinician: delete what is written in green before sending.” The green text is the English text and/or prompt to the template user.
(DOCX)

S2 File Clinician and caregiver surveys. The three surveys used in the study (first clinician survey, second clinician survey, and caregiver survey). The caregiver survey is presented in English and Spanish.
(DOCX)
