# Supplementary material for: The development of a technologic approach to improve access to individualized clinical documentation in caregivers’ preferred language
Source: PLOS Digit Health. 2026 Sep 11;5(9):e0001721. doi: 10.1371/journal.pdig.0001721 (PMC13568490; doi:10.1371/journal.pdig.0001721)
Supplement: Supplementary file 1 [file pdig.0001721.s001.docx]

**S1. Translated Evaluation Template.** Sample of template used for translating patient evaluation reports into Spanish from English.

Note. In the template, the user is prompted with the following message: “***Note to clinician: delete what is written in green before sending.” The green text is the English text and/or prompt to the template user.

[Patient's Name: @NAME@] Nombre del paciente: @NAME@

MRN: @MRN@

[Date of Visit: *DATE OF VISIT*] Fecha de la visita: ***DATE OF VISIT

[Visit Type] Tipo de visita

{Blank single:19197:: "[Bilingual Speech-Language Evaluation] Evaluación bilingüe del habla y lenguaje", "[Speech-Language Evaluation] Evaluación del habla y lenguaje", "[Bilingual Speech-Language Re-evaluation] Reevaluación bilingüe del habla y lenguaje", "[Fluency Evaluation] Evaluación de la fluidez", "[Fluency Re-evaluation] Reevaluación de la fluidez"}

**[Language of Testing] Idioma de la evaluación**

- [English] Inglés
- [Spanish via Qualified Bilingual Staff] Español con (este) personal bilingüe calificado
- [English and Spanish via Qualified Bilingual Staff] Español e inglés con (este) personal bilingüe calificado
- [Spanish via Interpreter] Español con un/a intérprete
- [English and Spanish via Interpreter] Español e inglés con un/a intérprete

**[Service History] Historial de servicios**

- [Past Related Services] Servicios relacionados anteriores
  - [Early intervention] Intervención temprana
  - [School-based speech therapy] Terapia del habla en la escuela
  - [Private speech therapy] Terapia del habla (privada)
  - [IEP or 504] Plan de Educación Individual (IEP) o Plan 504
  - [Behavior support] Apoyo conductual
- [Current Related Services] Servicios relacionados actuales
  - [Early intervention] Intervención temprana
  - [School-based speech therapy] Terapia del habla en la escuela
  - [Private speech therapy] Terapia del habla (privada)
  - [IEP or 504] Plan de Educación Individual (IEP) o Plan 504
  - [Behavior support] Apoyo conductual

[Impressions] Impresion diagnóstica

**Please see below for a list of impressions based on the recent speech-language evaluation, including parent and/or caregiver report and clinical observation.**

**A continuación, encontrará una lista de impresiones basadas en la reciente evaluación del habla y el lenguaje, incluido el informe de los padres y/o cuidadores y las observaciones clínicas.**

**[Diagnosis] Diagnóstico**

- [Mixed receptive-expressive language disorder] Trastorno mixto del lenguaje receptivo-expresivo
- [Delayed developmental milestones] Hitos retrasados del desarrollo
- [Expressive language disorder] Trastorno del lenguaje expresivo
- [Other symbolic dysfunctions] Otras disfunciones simbólicas
  - [Description: Deficits related to an organic or medical condition] Descripción: Deficiencias relacionadas a trastornos orgánicos o médicos
- [Phonological disorder] Trastorno fonológico
- [Articulation disorder] Trastorno de la articulación de sonidos
- [Social pragmatic communication disorder] Trastorno de la comunicación social pragmático
- [Childhood onset fluency disorder or Stuttering] Trastorno de la fluidez verbal de inicio en la infancia o tartamudez
- [Developmental language disorder with impairment of receptive and expressive language] Trastorno del desarrollo del lenguaje con deterioro del lenguaje receptivo y expresivo

**[Related Medical Diagnoses] Diagnósticos médicos relacionados**

- [Autism spectrum disorder] Trastorno del espectro autista
- [History of prematurity] Antecedentes de prematuridad
- [Developmental delay] Retraso del desarrollo
- [Global developmental delay] Retraso global del desarrollo
- [Genetic defect] Defecto genético
- [Conductive hearing loss] Pérdida auditiva conductiva
- [Sensorineural hearing loss] Pérdida auditiva neurosensorial

**Receptive Language**

**Lenguaje receptivo**

[The child's ability to understand what other people are saying.] La capacidad del niño/a para entender lo que los demás le dicen.

| *[Observed, or reported] Observado o reportado* | *[Emerging, inconsistent]*  *Emergente o inconsistente* | *[Expected, but not observed or reported]*  Esperado, pero no observado ni reportado |
| --- | --- | --- |
| *** | *** | *** |

**Expressive Language**

**Lenguaje expresivo**

[The child's ability to express their ideas and needs.] La capacidad del niño/a para expresar sus ideas y necesidades.

| *[Observed, or reported] Observado o reportado* | *[Emerging, inconsistent]*  *Emergente o inconsistente* | *[Expected, but not observed or reported]*  Esperado, pero no observado ni reportado |
| --- | --- | --- |
| *** | *** | *** |

**Social Communication**

**Comunicación social**

[The child's ability to communicate in social contexts (e.g., social interaction).] La capacidad del niño/a para comunicarse en entornos sociales (p.ej., interacciones sociales).

| *[Observed, or reported] Observado o reportado* | *[Emerging, inconsistent]*  *Emergente o inconsistente* | *[Expected, but not observed or reported]*  Esperado, pero no observado ni reportado |
| --- | --- | --- |
| *** | *** | *** |

**Play**

**Juego**

[Play is evaluated due to its close relationship with learning and cognition.] Se evalúa el juego debido a su estrecho vínculo con el aprendizaje y la cognición.

| *[Observed, or reported] Observado o reportado* | *[Emerging, inconsistent]*  *Emergente o inconsistente* | *[Expected, but not observed or reported]*  Esperado, pero no observado ni reportado |
| --- | --- | --- |
| *** | *** | *** |

**Speech Sound Production**

**Producción de los sonidos del habla**

[The child's ability to produce sounds in words and connected speech.] La capacidad del niño/a para producir sonidos en palabras y discurso.

- *******

**Intelligibility**

**Inteligibilidad**

[The child's ability to be understood by others.] La capacidad del niño/a de ser entendido por los demás.

- ***

**Speech Fluency (Early Childhood: 2 to 5 years)**

**Fluidez del habla (Primera infancia: 2 a 5 años)**

[The child's ability to talk smoothly with an appropriate rate of speech.] La capacidad del niño/a para hablar de manera fluida y con un ritmo del habla adecuado.

| *[Positive Prognostic Factors*  *(i.e., Patient does not present with these risk factors)]*  *Factores pronósticos positivos (i.e., el paciente no presenta estos factores de riesgo)* | *[Risk Factors for Continued Stuttering]*  *Factores de riesgo para la tartamudez persistente* |
| --- | --- |
| *** | *** |

**Speech Fluency (General)**

**Fluidez del habla (General)**

[The child's ability to talk smoothly with an appropriate rate of speech.] La capacidad del niño/a para hablar de manera fluída y a un ritmo adecuado del habla.

For clinician use only (clinician to add relevant bullets to chart and delete remaining):

Receptive Language

- [Demonstrate understanding a variety of age-appropriate vocabulary (e.g., common objects, familiar people, body parts)] Demuestra comprensión de una variedad de vocabulario apropiado para la edad (p.ej., objetos comunes, gente familiar, partes del cuerpo)
- [Demonstrate understanding of routine and highly familiar directions (e.g., clean up, high five, give me”)] Demuestra comprensión de instrucciones familiares y rutinarias (p.ej., limpiar/guardar los juguetes, dame los cinco/chócala, dámelo)
- [Demonstrate understanding of 1-step directions (e.g., “point to _”, “give me _”)] Demuestra comprensión de instrucciones de un solo paso (p.ej., “señala _”, “dame _”)
- [Demonstrate understanding of 2-step directions (e.g., “get your jacket and give it to mom”)] Demuestra comprensión de instrucciones de dos pasos (p.ej., “agarra tu chaqueta y dásela a mamá”)
- [Demonstrate understanding of directions containing locations (e.g., “put _ on your head”)] ”)] Demuestra comprensión de instrucciones con conceptos de ubicación (p.ej., “ponte __ en tu cabeza”)
- [Demonstrate understanding of the function of objects (e.g., “show me what you put on your foot”)] Demuestra comprensión de las funciones de los objetos (p.ej., “muéstrame lo que te pones en el pie”)
- [Demonstrate understanding of action commands (e.g., “jump”] Demuestra comprensión de instrucciones de acción (p.ej., “brinca”)
- [Demonstrate understanding 2-3 word phrases (e.g., ***)] ”)] Demuestra comprensión de frases de 2 a 3 palabras (p.ej., ***)
- [Demonstrate understanding age-appropriate WH-questions, including:] Demuestra comprensión de preguntas interrogativas adecuadas para la edad, incluyendo:
  - [Yes /no] øSí/No?
  - [What] øQué?
  - [Where] øDónde?
  - [What+doing] øQué está(s) haciendo?
  - [Who] øQuién?
  - [How many] øCuánto(s)?
- [Demonstrate understanding of pronouns (e.g., 'my/your')] Demuestra comprensión de los pronombres (p.ej., “mi/tu”)
- [Demonstrates understanding of complex questions (e.g., why, how)] Demuestra comprensión de preguntas complejas (p.ej., por qué, cómo)
- [Demonstrates understanding of inferential and/or prediction questions] Demuestra comprensión de preguntas inferenciales y/o de predicción
- [Demonstrates understanding of negation (e.g., “show me the baby who is *not* crying”)] Demuestra comprensión de la negación (p.ej., “muéstrame el bebé que *no* está llorando”)

Expressive Language Lenguage Expresivo

- [Express basic wants and needs through:] Expresa deseos y necesidades básicas a través de:
  - [Vocalizations] Vocalizaciones
  - [Body language] Lenguaje corporal
  - [Gestures/signs] Gestos/señas
  - [Words] Palabras
  - [Phrases/simple sentences] Frases/oraciones simples
  - [Anticipation/interpretation from caregivers] Anticipación/interpretación de los cuiadadores
- [Name familiar objects] Nombra objetos familiares
- [Name familiar actions] Nombra acciones familiares
- [Protest or rejects using a gesture] Protesta o rechaza con un gesto
- [Produce two-word phrases (e.g., agent+object, agent+verb, adjective+object)] Produce frases de dos palabras (p.ej., agente+objeto, agente+verbo, adjetivo+objeto)
- [Produce 3-4 word phrases] Produce frases de 3 a 4 palabras
- [Generate complete sentences (e.g., “he was hungry”)] Produce oraciones completas (p.ej., “el niño tenía hambre”)
- [Generate complex sentences (e.g., “he went to the store because he was hungry”)] Produce oraciones complejas (p.ej., “el niño fue a la tienda porque tenía hambre”)
- [Generate compound sentences (e.g., “he went to the store and bought some food”)] Produce oraciones compuestas (p.ej., “el niño fue a la tienda y compró algo de comida”)
- [Name location of objects] Nombra la ubicación de los objetos
- [Name colors] Nombra los colores
- [Answer age-appropriate WH-questions, including:] Responde preguntas interrogativas adecuadas para la edad, incluyendo:
  - [Yes /no] øSí/No?
  - [What] øQué?
  - [Where] øDónde?
  - [What+doing] øQué está(s) haciendo?
  - [Who] øQuién?
  - [How many] øCuánto(s)?
- [Ask age-appropriate questions, including:] Hace preguntas adecuadas para la edad, incluyendo:
  - [Yes /no] øSí/No?
  - [What] øQué?
  - [Where] øDónde?
  - [What+doing] øQué está(s) haciendo?
  - [Who] øQuién?
  - [How many] øCuánto(s)?
- [Generates jargon-like speech] Produce habla similar a la jerga
- [Uses specific vocabulary to tell a story or describe a picture scene] Usa vocabulario específico para contar una historia o describir una escena de imagen
- [Uses complete sentences to tell a story or describe a picture scene] Usa frases completas para contar una historia o describir una escena de imagen
- [Uses complex sentences to tell a story or describe a picture scene] Usa frases complejas para contar una historia o describir una escena de imagen
- [Uses prepositions (e.g., in, under, next to) to describe location] Usa preposiciones (p.ej., en, debajo de, al lado de) para describir la ubicación
- [Uses sequencing words (e.g., first, then, last, next) to retell or tell a story] Usa palabras de secuencia (p.ej., primero, entonces, por ˙ltimo, después) para contar o volver a contar una historia
- [Produces single-word utterances] Produce enunciados de una sola palabra
- [Communicates primarily through gestures and vocalizations] Se comunica principalmente con gestos y vocalizaciones
- [Communicates primarily through gestures] Se comunica principalmente con gestos
- [Communicates primarily through physical manipulation and anticipation of needs] Se comunica principalmente con manipulación física y la anticipación de necesidades
- [Imitates vocalizations] Imita vocalizaciones
- [Imitates words] Imita palabras
- [Imitates phrases] Imita frases

Social Communication Comunicación social

- [Use language for a variety of reasons (e.g., requesting, answering questions, making comments)] Usa el lenguaje por diversas razones (p.ej., pedir, responder preguntas, hacer comentarios)
- [Attend to shared materials] Presta atención a los materiales compartidos
- [Engage in appropriate eye contact/body orientation towards communication partners] Participa en el contacto visual /orientación corporal apropiados hacia el compañero de comunicación
- [Shift gaze in the following scenarios:] Cambia la mirada en los siguientes escenarios
  - [When making requests] Para pedir
  - [To see if play partners are watching what they are doing] Para ver si el compañero de juego está mirando lo que están haciendo
  - [To watch how play partners are interacting with materials] Para mirar cómo interact˙a el compañero de juego con los materiales
- [Engage in social moments (“check-ins”), such as:] Participa en momentos sociales (“chequeos”), como ser:
  - [Looking at others to see their reaction] Mirar a los demás para ver su reacción
  - [Smiling/laughing during unexpected actions] Sonreír/reírse durante acciones inesperadas
- [Point to share and/or show something to someone else] Señalar para compartir y/o mostrarle algo a alguien más
- [Follow a point by looking in that direction] Seguir un punto mirando en esa dirección [Engage in early turn-taking through back-and-forth play (e.g., ball play, imitating actions)] Toma su turno de manera esperada para la edad, mediante juegos con turnos (p.ej., juego con pelota, imitar acciones)
- [Initiate to make requests for objects/actions, make comments, ask questions] Tiene iniciativa para pedir objetos/acciones, hacer comentarios, hacer preguntas
- [Respond to others when called, asked questions, or given directions] Responde a los demás cuando lo llaman, cuando le hacen preguntas, o cuando le dan indicaciones
- [Engage in emerging conversational turn-taking (e.g., responding to questions/prompts)] Participa en turnos de conversación emergentes (p. ej., responde preguntas/indicaciones)
- [Maintain a topic of conversation (e.g., 2-3+ turns on the same topic)] Mantiene el tema de conversación (p.ej., 2 a 3+ turnos del mismo tema)

Play Juego

- [Demonstrate interest in exploring new toys and/or playing with toys in different ways] Demuestra interés en explorar juguetes nuevos y/o en jugar con juguetes de diferentes maneras
- [Imitating new/different play] Imita el juego nuevo o diferente
- [Expand on play given directions and/or models] Amplía el juego con instrucciones y/o ejemplos
- [Engage in functional play (e.g., using objects as they are intended)] Participa en juegos funcionales (p.ej., usa objetos para lo que están destinados)
- [Engage in pretend play (e.g., feeding bear, putting a toy bear to bed)] Participa en juegos de simulación (p.ej., darle de comer a un osito de peluche; poner el osito de peluche a dormir)
- [Engage in cooperative play (e.g., playing together)] Participa en juegos cooperativos (p.ej., jugar juntos)

Speech Sound Production Producción de los sonidos del habla

- [@FNAME@ had difficulty producing the following sounds in words: ***] @FNAME@ tuvo dificultad para producir los siguientes sonidos en palabras: ***
- [@FNAME@ had difficulty producing the following sounds in sentences: ***] @FNAME@ tuvo dificultad para producir los siguientes sonidos en frases: ***

Intelligibility Inteligibilidad

- [Understood by immediate family members (e.g., parents, caregivers, siblings)] Entendido por la familia inmediata (p.ej., padres, cuidadores, hermanos)
- [Understood by extended family members (e.g., cousins, aunts, uncles)] Entendido por la familia extendida (p.ej., primos, tíos)
- [Understood by peers] Entendido por los compañeros/amigos
- [Understood by teachers or daycare providers] Entendido por los maestros o proveedores de la guardería
- [Understood by strangers] Entendido por personas desconocidas

Speech Fluency (Early Childhood: 2 to 5 years) Fluidez del habla (Primera infancia: 2 a 5 años)

*Risk Factors Factores de riesgo*

- [Age at onset (children who start stuttering after 35-months may be at higher risk of continued stuttering)] Edad de inicio (los niños/as que empiezan a tartamudear después de los 35 meses pueden correr mayor riesgo de tartamudez persistente)
- [Consistency (children whose stutter-like disfluencies decrease within the first year are less likely to experience continued stuttering)] Consistencia (los niños/as cuyos tartamudeos disminuyen dentro del primer año tienen menos probabilidad de tartamudez persistente)
- [Temperament (children who are more reactive to their stuttering may have difficulty regulating their reactions to stuttering)] Temperamento (los niños/as que son más reactivos a su tartamudez pueden tener dificultad para regular sus reacciones a la tartamudez)
- [Family history (children with family history of stuttering are at a higher risk of continued stuttering)] Antecedentes familiares (los niños/as con antecedentes familiares de tartamudez corren mayor riesgo de tartamudez persistente)
- [Sex (female children are less likely to experience continued stuttering than male children)] Sexo (las niñas son menos propensas a la tartamudez persistente que los niños)
- [Time since onset (most children stop stuttering within the first year post-onset. The longer a child stutters, the higher the risk of continued stuttering)] Tiempo desde el inicio/aparición (la mayoría de los niños/as dejan de tartamudear durante el primer año. Cuanto más tiempo tartamudea el niño/a, mayor será el riesgo de tartamudez persistente)
- [Disfluency types (children who exhibit more stutter-like disfluencies than typical disfluencies may be at higher risk of continued stuttering)] Tipos de disfluencias (los niños/as que demuestran más disfluencias parecidas al tartamudeo que las disfluencias típicas, podrían tener mayor riesgo de tartamudez persistente)

Speech Fluency (General) Fluidez del habla (General)

- [@FNAME@ exhibited the following types of stutter-like disfluencies:] @FNAME@ presentó los siguientes tipos de tartamudez:
  - [Blocks (e.g., ***)] Bloqueos (p.ej., ***)
  - [Prolongations (e.g., ***)] Prolongaciones (p.ej., ***)
  - [Single-syllable whole word repetitions (e.g., ***)] Repetición de palabra entera de una sílaba (p.ej., ***)
  - [Part-word repetitions (e.g., ***)] Repetición de una parte de la palabra (p.ej., ***)
  - [Sound repetitions (e.g., ***)] Repetición de sonido (p.ej., ***)
- [@FNAME@ exhibited the following types of typical disfluencies:] @FNAME@ presentó los siguientes tipos de disfluencias típicas:
  - [Interjections (e.g., ***)] Interjecciones (p.ej., ***)
  - [Reformulations (e.g., ***)] Reformulaciones (p.ej., ***)
  - [Multi-syllable whole-word repetitions (e.g., ***)] Repetición de palabra multisílaba entera (p.ej., ***)
- [@FNAME@ exhibited the following types of atypical disfluencies (i.e., disfluencies that are not stutter-like, but do not fall under the category of typical):] @FNAME@ presentó los siguientes tipos de disfluencias atípicas (i.e., disfluencias que no son tartamudeos, pero tampoco entran en la categoría de lo típico)
  - [Mid-word breaks (e.g., ***)] Salto en mitad de palabra (p.ej., ***)
  - [Final-sound repetitions (e.g., ***)] Repetición del sonido final (p.ej., ***)
  - [Final-sound prolongations (e.g., ***)] Prolongación del sonido final (p.ej., ***)
  - [Mid-word repetition (e.g., ***)] Repetición en mitad de la palabra (p.ej., ***)
  - [Sound insertion (e.g., ***)] Inserción de sonido (p.ej., ***)
- [@FNAME@ exhibited the following concomitant behaviors:] @FNAME@ presentó los siguientes comportamientos concomitantes:
  - [Eye blinking] Parpadeo de los ojos
  - [Reduced eye contact] Contacto visual reducido
  - [Tapping fingers] Tamborileo de los dedos
  - [Clearing throat] Aclararse la garganta
  - [Heavy breathing] Respiración pesada
  - [Turning head] Girar la cabeza
- [Based on the results of a formal questionnaire and patient report, it was determined that stuttering has a {mild, mild-to-moderate, moderate, moderate-to-severe, severe} impact on @FNAME@'s life.] Basado en los resultados del cuestionario formal y del reporte de los padres, se determinó que la tartamudez tiene un impacto {[mild] leve, [mild-to-moderate] leve a moderado, [moderate] moderado, [moderate-to-severe] moderado a profundo, [severe] profundo} en la vida de @FNAME@.

Recomendaciones [Recommendations]

*Tratamiento* [Treatment]

{Blank single:19197:: "Se recomienda la terapia del habla y lenguaje con el Departamento del Habla y Lenguaje de CLINIC. [Treatment with the CLINIC Speech Department is recommended]", "No se recomienda la terapia del habla y lenguaje con el Departamento del Habla y Lenguaje de CLINIC. [Treatment at the CLINIC Speech Department is not recommended]"}

[Treatment will target] *El tratamiento se dirigirá a estos objetivos:*

{Blank multiple:19196::"[Expressive language goals] Los objetivos del lenguaje expresivo", "[Receptive language goals] Los objetivos del lenguaje receptivo", "[Social communication goals] Los objetivos de la comunicación social", "[Speech sound production goals] Los objetivos de la producción de los sonidos del habla", "[Intelligibility goals] Los objetivos de la inteligibilidad", "[Stuttering goals] Los objetivos de la tartamudez", "[Fluency goals] Los objetivos de la fluidez"}

**[Suggested Goals and Objectives] Metas y objetivos propuestos**

**[Long Term Goal #***:** @FNAME@ will improve pragmatic and play skills]

**Objetivo a largo plazo #***:** @FNAME@ mejorará las destrezas pragmáticas y de juego

- [@FNAME@ will consistently respond to @HIS@ name by looking at an adult in 80% of elicited opportunities in play.] ‎@FNAME@ responderá de manera consistente a @SU@ nombre mirando a un adulto en el 80 % de las ocasiones que se le presenten en el juego. ‎
- [@FNAME@ will respond to social interaction by imitating actions and facial expressions, sharing toys, and participating in turn-taking play in 80% of opportunities.] @FNAME@ responderá a la interacción social, imitando acciones y expresiones faciales, compartiendo juguetes, y participando en juegos por turnos, en el 80 % de las ocasiones
- [@FNAME@ will increase initiation of social interaction by initiating play exchange in at least 10 opportunities in one play activity over 3 consecutive sessions.] @FNAME@ aumentará la toma de iniciativa de interacciones sociales, iniciando el intercambio de juegos en al menos 10 ocasiones en una actividad de juego, durante 3 sesiones consecutivas.
- [@FNAME@ will demonstrate communicative intent when expressing needs (e.g., requesting, rejecting, asking for assistance) by making eye-contact, tapping, or calling an adult's name 80% of the time.] @FNAME@ demostrará una intención comunicativa cuando exprese sus necesidades (p. ej., pedir, rechazar, pedir ayuda) al hacer contacto visual, o tocar o llamar a un adulto por su nombre, el 80 % de las veces.
- [@FNAME@ will demonstrate functional use of objects when given toys (e.g., spoon, cup, ‎brush, phone, etc.) initially in imitation progressing to independent use in 80% of opportunities.] @FNAME@ demostrará el uso funcional de los objetos cuando se le den juguetes (por ejemplo, una cuchara, una taza, un cepillo, un teléfono, etc.) inicialmente en forma de imitación, progresando a un uso independiente en el 80 % de las ocasiones.
- [@FNAME@ will demonstrate representational play by utilizing common objects appropriately to engage in play toward a doll (e.g., feeding a doll with a spoon, putting a doll to bed, washing a doll, etc...) initially in imitation progressing to independent use in 80% of opportunities.] @FNAME@ demostrará el juego representativo al utilizar objetos comunes de manera apropiada, al participar en juegos con una muñeca (p. ej., alimentar la muñeca con una cuchara, acostar la muñeca, lavar la muñeca, etc.), inicialmente en forma de imitación, y progresando a un uso independiente en el 80% de las ocasiones.
- [@FNAME@ will demonstrate 1-2 step symbolic play sequences enacting familiar routines and actions (i.e., eating, drinking, washing hands, taking a bath, etc..) given an initial model progressing to independent play in 80% of opportunities.] @FNAME@ demostrará secuencias de juego simbólico de 1 a 2 pasos, que representan rutinas y acciones familiares (es decir, comer, beber, lavarse las manos, bañarse, etc.) con un modelo inicial que progresa hacia el juego independiente en 80 % de las ocasiones.

**[Long Term Goal #***:** @FNAME@ will improve receptive language skills]

**Objetivo a largo plazo #***:** @FNAME@ mejorará las destrezas del lenguaje receptivo

- [@FNAME@ will consistently respond to @HIS@ name and to “no” by looking at adult in 80% of elicited opportunities in play.] @FNAME@ responderá de manera consecuente a @SU@ nombre y al "no”, mirando al adulto en el 80 % de las ocasiones que se le presenten en el juego.
- [@FNAME@ will follow simple commands (e.g., give me, stop, go, sit down, clean-up) in play tasks in 80% of opportunities.‎] @FNAME@ seguirá órdenes simples (p. ej., dame, para, ve, siéntate, limpia) en tareas de juego en el 80 % de las ocasiones.‎
- [@FNAME@ will identify six body parts and/or clothing items in 80% of opportunities.‎‎] @FNAME@ identificará seis partes del cuerpo y/o prendas de vestir en el 80 % de las ocasiones
- [@FNAME@ will identify a variety of familiar objects in play and in pictures, as well as in early categories (i.e., body parts, clothing, animals, food, vehicles, etc...) in 80% of opportunities.] @FNAME@ identificará una variedad de objetos familiares en el juego y en las imágenes, así como en las categorías iniciales (partes del cuerpo, ropa, animales, alimentos, vehículos, etc.), en el 80 % de las ocasiones.
- [@FNAME@ will follow one-step commands such as “give me, stop, go, sit” with early prepositions “in, on, up, and down” in play tasks in 80% of opportunities.‎‎] @FNAME@ seguirá indicaciones de un solo paso, como: "dame, detente, ve, siéntate", con preposiciones iniciales: "adentro, arriba, abajo", en tareas de juego, en el 80 % de las ocasiones.‎‎
- [@FNAME@ will demonstrate comprehension of early action words with manipulatives and identify these actions in pictures in 80% of elicited opportunities.‎] @FNAME@ demostrará comprensión de palabras iniciales de acción con manipulativos, e identificará estas acciones en imágenes en el 80 % de las ocasiones que se le presenten.‎
- [@FNAME@ will follow novel and two-step commands containing a variety of nouns, verbs, and prepositions in structured tasks with 80% accuracy given verbal and visual models.] @FNAME@ seguirá indicaciones novedosas y de dos pasos, que contengan una variedad de sustantivos, verbos y preposiciones, en tareas estructuradas, con un

80 % de precisión al recibir modelos verbales y visuales.

- [@FNAME@ will follow directions with early linguistic concepts, such as those related to location (up/down, in/out, on/under), quantity (one, all) and size (big, small), with 80% accuracy.] @FNAME@ seguirá instrucciones con conceptos lingüísticos iniciales, como los relacionados con la ubicación (arriba/abajo, adentro/afuera, arriba/abajo), cantidad (uno, todo) y tamaño (grande, pequeño), con un 80 % de precisión.
- [@FNAME@ will accurately respond to simple questions (i.e., yes/no, where, what + doing, what, who) about things in the immediate environment with 80% accuracy.]‎ @FNAME@ responderá con precisión a preguntas simples (es decir: sí/no, dónde, qué + haciendo, qué, quién) sobre cosas en el entorno inmediato, con un 80 % de precisión.‎

**[Long Term Goal #***:** @FNAME@ will improve expressive language skills]

**Objetivo a largo plazo #***:** @FNAME@ mejorará las destrezas del lenguaje expresivo

- [@FNAME@ will expand use of gesture and modified picture exchange in combination with vocalization as a means of communicating requests (e.g., pointing, conventional gestures, picture icons in combination with known and/or taught words) in 80% of opportunities.‎] @FNAME@ ampliará el uso de gestos e intercambio de imágenes modificadas en combinación con la vocalización como medio para comunicar solicitudes (p. ej., señalar, gestos convencionales, íconos de imágenes en combinación con palabras conocidas y/o enseñadas), en el 80 % de las ocasiones.‎
- [@FNAME@ will expand use of communicative gestures (e.g., clapping, waving "hi" and "bye", pointing) initially in imitation and progressing to independent use 80% of the time.]

@FNAME@ ampliará el uso de gestos comunicativos (p. ej., aplaudir, saludar con la mano "hola" y "adiós", señalar), inicialmente en forma de imitación y progresando hacia un uso independiente, el 80 % del tiempo

- [@FNAME@ will sign or verbalize immediate needs to request and reject items or activities, and to ask for help in 80% of opportunities.‎] @FNAME@ utilizará la seña o verbalizará necesidades inmediatas para solicitar y rechazar artículos o actividades y pedir ayuda en el 80% de las ocasiones.‎
- [@FNAME@ will imitate environmental and animal sounds in play tasks in 80% of opportunities.] ‎@FNAME@ imitará los sonidos ambientales y de animales en las tareas de juego en el 80 % de las ocasiones. ‎
- [@FNAME@ will imitate and, later, independently name familiar agents (people, animals, etc...) and objects (body parts, toys, foods, furniture, etc...) in the environment, in pictures, and in early categories with 80% accuracy.‎] @FNAME@ imitará y, posteriormente, nombrará de forma independiente agentes familiares (personas, animales, etc) y objetos (partes del cuerpo, juguetes, alimentos, muebles, etc) en el entorno, en imágenes y en las categorías iniciales, con un 80% de precisión.‎
- [@FNAME@ will request and reject using multi-word utterances with a specific label (e.g., *I want + ____; clean up + _____; all done + ______)* in structured activities and eventually spontaneously 80% of the time.] @FNAME@ solicitará y rechazará el uso de expresiones de varias palabras con una etiqueta específica (p. ej., quiero + ____; a limpiar + _____; terminé + ______) en actividades estructuradas y, finalmente, de forma espontánea, el 80 % del tiempo.
- [@FNAME@ will imitate word combinations describing early semantic relations (e.g., action + object, agent + action) and eventually produce these spontaneously in 80% of opportunities.‎] @FNAME@ imitará combinaciones de palabras que describen relaciones semánticas iniciales (p. ej., acción + objeto, agente + acción) y, finalmente, las producirá espontáneamente, en el 80 % de las ocasiones.‎
- [@FNAME@ will label a variety of early actions in pictures and in the environment and begin to use them in word combinations in 80% of opportunities.] @FNAME@ etiquetará una variedad de acciones iniciales en imágenes y en el entorno, y comenzará a usarlas en combinaciones de palabras, en el 80 % de las ocasiones.
- [@FNAME@ will inquire of object names with “what is it?” and actions with “what is s/he doing?” 80% of the time.] @FNAME@ preguntará sobre los nombres de los objetos con "øqué es?", y acciones con "øqué está haciendo?", 80% del tiempo.
- [@FNAME@ will state @HIS@ name upon request in 80% of opportunities.] @FNAME@ indicará @SU@ nombre cuando se le solicite, en el 80 % de las ocasiones.

**[Long-Term Goal ***:** @FNAME@'s *** will use strategies to encourage effective communication and prevent or avoid negative reactions to stuttering.]

**Objetivo a largo plazo** ***: @FNAME@ *** utilizará estrategias para fomentar una comunicación eficaz y prevenir o evitar reacciones negativas a la tartamudez.

- [@FNAME@'s *** will generate five factors that can contribute to disfluencies including child factors, factors in the communicative environment, and in the general environment and systematically identify those factors that apply to @FNAME@ in {Blank single:19197::"his","her"} daily life.] El *** de @FNAME@ generará cinco factores que pueden contribuir a las disfluencias, incluyendo: factores infantiles, factores en el entorno comunicativo, y en el entorno general, e identificará sistemáticamente los factores que se aplican a la vida diaria de @FNAME@ {Blank single: 19197::
- [@FNAME@'s *** will identify 4-5 factors in @FNAME@'s home environment that could contribute to increased stuttering.] El *** de @FNAME@ identificará 4 ó 5 factores en el entorno del hogar de @FNAME@ que podrían contribuir a un aumento de la tartamudez.
- [@FNAME@'s *** will identify five strategies to reduce negative reactions to stuttering and facilitate a less-demanding communicative environment and employ identified techniques in sessions in 90% of opportunities given clinician feedback on parent-child interaction. Activities directly working on enhancing @FNAME@'s speech fluency may be added if necessary, such as modeling turn taking with easy relaxed speech as well as delayed response to increase pause time in speech.] El *** de @FNAME@ identificará cinco estrategias para reducir las reacciones negativas a la tartamudez, y facilitará un entorno comunicativo menos exigente, empleando las técnicas identificadas en las sesiones, en el 90 % de las ocasiones que se presenten, con comentarios del Clínico sobre la interacción entre padres e hijos. Si es necesario, se pueden agregar actividades que mejoren directamente la fluidez del habla de @FNAME@, como modelar la toma de turnos, con un discurso sencillo y relajado, así como respuestas demoradas, para aumentar el tiempo de pausa en el habla.

**[Long-Term Goal # ***: Improve expressive spoken language skills.**‎]

**Objetivo a largo plazo # ***:** Mejorar las destrezas expresivas del lenguaje hablado. ‎

- [@FNAME@ will expand his functions of communication (increase requests for actions/objects and cessation, increase use of commenting on his immediate environment, and increase use of greetings/social language) in 80% of opportunities.‎] @FNAME@ ampliará sus funciones de comunicación (aumento y cese de las solicitudes de acción/objetos, aumento en el uso de comentarios sobre su entorno inmediato y aumento en el uso de saludos/lenguaje social), en el 80% de las ocasiones.‎
- [@FNAME@ will increase natural imitation and gradually, spontaneous use of single words/word approximations reflecting early semantic concepts in 80% of opportunities. Emphasis should be on developing core vocabulary of relational words (those easily combined with others) and substantive (specific labels) words meaningful to @HIS@ daily life.] ‎@FNAME@ aumentará la imitación natural y, gradualmente, el uso espontáneo de palabras sueltas/aproximaciones de palabras, que reflejen conceptos semánticos iniciales, en el 80 % de las ocasiones. Se debe hacer hincapié en el desarrollo del vocabulario básico de palabras relacionales (aquellas que se combinan fácilmente con otras) y palabras sustantivas (etiquetas específicas) significativas para @SU@ vida diaria.
- [@FNAME@ will increase his use of specific terms to designate objects, actions, and events in the context of experiential and play activities with 80% accuracy.]‎ @FNAME@ aumentará el uso de términos específicos para designar objetos, acciones y eventos en el contexto de actividades experienciales y de juego, con un 80 % de precisión.‎
- [@FNAME@ will increase his use of the a variety of verb tenses including present progressive, copular verbs, past verb tense, and future tense through the sentence level with 80% accuracy.‎‎] @FNAME@ incrementará el uso de una variedad de tiempos verbales, incluyendo el presente progresivo, los verbos copulares, el tiempo verbal pasado y el tiempo futuro, a lo largo del nivel de la oración, con un 80 % de precisión.‎‎
- [@FNAME@ will increase his use of subject-verb-object sentences, articles and prepositions and early conjunctions in structured activities and spontaneous conversation with 80% accuracy.] ‎‎@FNAME@ incrementará el uso de oraciones sujeto-verbo-objeto, artículos y preposiciones y conjunciones iniciales, en actividades estructuradas y en conversaciones espontáneas, con un 80% de precisión. ‎‎
- [@FNAME@ will use *** word utterances in structured activities and spontaneous conversation with 80% accuracy.]‎‎ @FNAME@ utilizará ***expresiones verbales en actividades estructuradas y conversaciones espontáneas, con un 80 % de precisión.‎‎
- [@FNAME@ will increase @HIS@ use of pronouns (personal, objective, possessive) in structured and unstructured activities with 80% accuracy.‎‎] @FNAME@ aumentará @SU@ uso de pronombres (personal, objetivo, posesivo) en actividades estructuradas y no estructuradas, con un 80 % de precisión.‎‎
- [@FNAME@ will respond to wh-questions in structured activities and spontaneous conversation with 80% accuracy.‎‎] @FNAME@ responderá preguntas interrogativas en actividades estructuradas y en conversaciones espontáneas, con un 80 % de precisión.‎‎
- [@FNAME@ will initiate conversation in structured activities and spontaneous conversation with adults and peers in 80% of opportunities.‎‎] @FNAME@ iniciará conversaciones en actividades estructuradas y conversaciones espontáneas con adultos y compañeros, en el 80% de las ocasiones.

**[Long-Term Goal # *****: Demonstrate age appropriate language comprehension skills with family, peers, and clinicians in all communicative settings.‎]

**Objetivo a largo plazo # ***:** Demostrar destrezas de comprensión lingüística adecuadas para la edad, con la familia, los compañeros y los clínicos, en todos los entornos comunicativos. ‎

- [@FNAME@ will demonstrate ability to follow 1-2 step directions during play or familiar routines in both structured and unstructured environments with 90% accuracy.] @FNAME@ demostrará capacidad de seguir instrucciones de 1 a 2 pasos durante el juego o rutinas familiares, en entornos estructurados y no estructurados, con un 90 % de precisión. ‎
- [@FNAME@ will exhibit comprehension of basic concepts (including identification of common objects, body parts, prepositions, action words, and quantity) in both structured and unstructured environments with 90% accuracy.‎] @FNAME@ demostrará comprensión de conceptos básicos (incluida la identificación de objetos comunes, partes del cuerpo, preposiciones, palabras de acción y cantidad) en entornos estructurados y no estructurados, con un 90 % de precisión.‎
- [@FNAME@ will respond appropriately to age appropriate WH- questions with fading cues in both structured and unstructured environments with 90% accuracy.‎] @FNAME@ responderá adecuadamente a las preguntas interrogativas adecuadas para la edad, con señales decrecientes, en entornos estructurados y no estructurados, con un 90 % de precisión.‎
- [@FNAME@ will increase his understanding of specific terms to designate objects, actions, and events in the context of play activities with 90% accuracy‎.] @FNAME@ aumentará la comprensión de términos específicos para designar objetos, acciones y eventos, en el contexto de actividades de juego, con un 90 % de precisión.
- [@FNAME@ will increase understanding of early grammatical forms ( present progressive, plurals, auxiliary verbs) in both structured and unstructured environments with 90% accuracy.‎] @FNAME@ aumentará la comprensión de las formas gramaticales iniciales (presente progresivo, plurales, verbos auxiliares) en entornos estructurados y no estructurados, con un 90 % de precisión.‎

**[Long-Term Goal # ***:** Improve speech intelligibility in connected speech across settings and communication partners.]

**Meta a largo plazo n.∞ ***:** Mejorar la inteligibilidad del habla, en el habla conectada, en forma generalizada en distintos entornos y distintos compañeros de comunicación. ‎‎

- [@FNAME@ will demonstrate a reduction and elimination of the developmentally inappropriate phonological processes of final/medial consonant deletion, unstressed syllable deletion, stopping, fronting, vowel deviations, gliding and consonant cluster reductions/substitutions with fading prompts in sentences and in connected speech to 10 % occurrence.‎] @FNAME@ demostrará una reducción y eliminación de los procesos fonológicos inapropiados del desarrollo: eliminación de consonantes finales/mediales, eliminación de sílabas no acentuadas, detenciones, anteriorizaciones, desviación de vocales, deslizamiento y reducciones/sustituciones de grupos de consonantes, con indicaciones decrecientes, en oraciones y en el habla continua, a un 10 % de las ocurrencias.‎
- [@FNAME@ will produce the following sounds with 80% accuracy at the word level: ***.] @FNAME@ producirá los siguientes sonidos con un 80 % de precisión a nivel de palabra: ***.
- [@FNAME@ will produce the following sounds with 80% accuracy at the sentence level: ***.] @FNAME@ producirá los siguientes sonidos con un 80 % de precisión a nivel de oración: ***.
- [@FNAME@ will produce the following sounds with 80% accuracy at the conversational level: ***.] @FNAME@ producirá los siguientes sonidos con un 80 % de precisión a nivel de conversación: ***.
- [@FNAME@ will Increase self-monitoring and discrimination of productions within the word level to conversational speech with 90% accuracy.]‎ @FNAME@ aumentará el autocontrol y discriminación de sonidos del habla, dentro del nivel palabra al nivel de habla conversacional, con un 90 % de precisión.‎

**[Prognosis] Pronóstico**

- [Favorable] Favorable
- [Poor] Deficiente
- [Good] Bueno
- [Fair] Regular
- [Guarded] Reservado
- [Indeterminate] Indeterminado

**[Positive Prognostic Indicators] Indicadores pronósticos positivos**

- [Strong baseline functioning] Sólido funcionamiento de base
- [Recency of onset] Fecha más reciente de aparición/inicio
- [Strong participation and/or response to treatment] Sólida participación y/o respuesta al tratamiento
- [Limited co-morbidities] Comorbilidades limitadas
- [Improvement to date] Mejoría hasta la fecha
- [Supportive family/social environment] Entorno familiar/social favorable
- [Minimal overall disease burden] Mínima carga general de la enfermedad
- [Motivation/awareness/insight] Motivación/conciencia/discernimiento
- [Age] Edad

**[Negative Prognostic Indicators] Indicadores pronósticos negativos**

- [Underlying comorbidities] Comorbilidades subyacentes
- [Limited progress to date] Limitado progreso hasta la fecha
- [Age] Edad
- [Extensive disease burden] Excesiva carga de la enfermedad
- [Behavioral/cognitive limitations] Limitaciones conductuales/cognitivas
- [Severity of impairment] Gravedad del impedimento
- [Limited endurance or response to treatment] Mínima tolerancia o respuesta al tratamiento
- [Complicated hospital course] Curso hospitalario complicado
- [Limited social supports] Limitado apoyo social

**[Observations That Warrant Further Investigation] Observaciones que justifican una mayor investigación**

- [Behavior challenges] Dificultades del comportamiento
- [Inconsistent or decreased attention] Atención reducida o inconsistente
- [Social differences] Diferencias sociales
- [Reduced eye contact and/or joint attention] Atención conjunta y/o contacto visual reducidos
- [Parent concerns] Preocupaciones de los padres
- [Suspected motor difficulties] Presuntas dificultades motoras
- [Suspected structural differences in the oral mechanism] Presuntas diferencias estructurales en el mecanismo vocal
- [Suspected differences in sensory needs] Presuntas diferencias en las necesidades sensoriales

**[RECOMMENDATIONS] RECOMENDACIONES**

- [A **short term period of therapy services of up to 12 sessions 1x weekly for 50 minutes** is recommended at the {Blank single: 19197:: CLINIC", "CLINIC2", "CLINIC3}. Discussed scheduling with parents. The family will be contacted when a slot is open.] Se recomienda un período corto de servicios de terapia de hasta 12 sesiones, 1 vez por semana, por 50 minutos, en {Blank single: 19197:: CLINIC", "CLINIC2 ", "CLINIC3”} Se habló con los padres de agendar cita. Se contactará a la familia cuando haya un espacio disponible.

[Outside Referrals] *Remisiones externas*

{Blank multiple:19196::"[A school-based speech/language evaluation is recommended. See attached instructions] Se recomienda una evaluación escolar del habla y lenguaje. Se adjuntan instrucciones.", "[An audiological evaluation is recommended. Evaluating clinician will assist with referral.] Se recomienda una evaluación de la audición. La logopeda que hizo esta evaluación les ayudará con la remisión del paciente.", "[A neuropsychological evaluation is recommended. Evaluating clinician will assist with referral.] Se recomienda una evaluación neuropsicológica. La logopeda que hizo esta evaluación les ayudará con la remisión del paciente.", "[An Early Intervention referral is recommended. Evaluating clinician will assist with referral.] Se recomienda la remisión a Intervención Temprana. La logopeda que hizo esta evaluación les ayudará con la remisión del paciente", "[An occupational therapy evaluation is recommended. Evaluating clinician will assist with referral.] Se recomienda una evaluación de la terapia ocupacional. La logopeda que hizo esta evaluación les ayudará con la remisión del paciente", "[A written language evaluation is recommended through the CLINIC Speech Language Department. Evaluating clinician will assist with referral] Se recomienda una evaluación del lenguaje escrito a través del Departamento del Habla y Lenguaje de CLINIC. La logopeda que hizo esta evaluación les ayudará con la remisión del paciente.", "[A feeding evaluation is recommended through the CLINIC Speech Language Department. Evaluating clinician will assist with referral.] Se recomienda una evaluación de la alimentación. La logopeda que hizo esta evaluación les ayudará con la remisión del paciente.", "[A physical therapy evaluation is recommended. This clinician will assist with referral.] Se recomienda una evaluación de fisioterapia. La logopeda que hizo esta evaluación les ayudará con la remisión del paciente.", "[Recommendations to be discussed further with clinician and interpreter] Las recomendaciones se discutirán más a fondo con la logopeda y un intérprete."}

[Behavior] Conducta

{Blank single:19197:: "[@FNAME@ sat and attended during formal and informal testing.] @FNAME@ se sentó y puso atención durante los exámenes formales y informales.", "[@FNAME@ sat and had some difficulty attending during formal and informal testing.] @FNAME@ se sentó y tuvo dificultad para poner atención durante los exámenes formales y informales.", "[@FNAME@ did not sit or attend during formal and informal testing.] @FNAME@ no se sentó ni puso atención durante los exámenes formales y informales.", "[Behavior to be discussed further with clinician and interpreter] La conducta se discutirá más a fondo con la logopeda y un intérprete."}

[Tests Administered] Pruebas administradas

{Blank multiple:19196::"[Standardized language assessment] Evaluación estandarizada del lenguaje", "[Formal, yet not standardized, language assessment] Evaluación formal, pero no estandarizada, del lenguaje", "[Language sample analysis] Análisis del ejemplo del lenguaje", "[Parent interview] Entrevista con los padres", "[Standardized speech sound production assessment] Evaluación estandarizada de la producción de los sonidos del habla", "[Formal, yet not standardized, speech sound production assessment] Evaluación formal, pero no estandarizada, de la producción de los sonidos del habla", "[Clinical observation] Observaciones clínicas"}

[Treatment will target] *El tratamiento tendrá como objetivos:*{Blank multiple:19196::"[Expressive language goals] Objetivos del lenguaje expresivo", "[Receptive language goals] Objetivos del lenguaje receptivo", "[Social communication goals] Objetivos de la comunicación social", "[Speech sound production goals] Objetivos de la producción de los sonidos del habla", "[Intelligibility goals] Objetivos de la inteligibilidad", "[Stuttering goals] Objetivos de la tartamudez", "[Fluency goals] “Objetivos de la fluidez", [Play goals] Objetivos del juego”}

[@FNAME@'s family is encouraged to contact me with any questions or concerns at the CLINIC Department of Speech, Language and Swallowing Disorders at *PHONE NUMBER* or via e-mail at *EMAIL ADDRESS*.]

Para cualquier duda o pregunta que tenga, recomendamos a la familia de @FNAME@ ponerse en contacto conmigo en el Departamento del Habla, Lenguaje, Trastornos de Deglución de CLINIC, y puede llamarme al ***PHONE NUMBER o enviarme un correo electrónico al ***EMAIL ADDRESS.

[Regards,] Atentamente,

***CLINICIAN SIGNATURE

***CLINICIAN NAME

[Pediatric Speech-Language Pathologist] Logopeda Pediátrica

{Blank single:19197:: "CLINIC1", "CLINIC2", "CLINIC3"}
