# Supplementary material for: The development of a technologic approach to improve access to individualized clinical documentation in caregivers’ preferred language
Source: PLOS Digit Health. 2026 Sep 11;5(9):e0001721. doi: 10.1371/journal.pdig.0001721 (PMC13568490; doi:10.1371/journal.pdig.0001721)
Supplement: Supplementary file 2 [file pdig.0001721.s002.docx]

**S2 File. Clinician and Caregiver Surveys.** The three surveys used in the study (first clinician survey, second clinician survey, and caregiver survey). The caregiver survey is presented in English and Spanish.

Note:

- Indicates a single-response question
- Indicates a multiple-select question

**First Clinician Survey**

How many years have you been practicing as a certified speech-language pathologist?

- 1-5
- 6-10
- 11-20
- 21-30
- 31+

Are you ASHA-certified?

- Yes
- No
- Unsure

Are you a licensed SLP in the state of Massachusetts?

- Yes
- No
- Unsure

What is your primary language?

- English
- Spanish
- Portuguese
- Arabic
- Other

How do you currently communicate evaluation results to caregivers who primarily speak/read Spanish?

- I orally translate the evaluation results to the caregiver(s) during a phone call, video call, and/or in-person appointment.
- I expect the caregiver(s) family and/or friends to translate the results for them.
- I request a formally translated version of the evaluation report to be sent to the parent.
- I provide an oral summary of the evaluation results for the caregiver(s) during a phone call, video call, and/or in-person appointment.
- I do not know
- I prefer not to answer
- Other (describe below)

How do you currently communicate evaluation results to caregivers who primarily speak/read Spanish? ________________________________________________________________

How do you currently communicate evaluation recommendations to caregivers who primarily speak/read Spanish?

- I orally translate the evaluation recommendations to the caregiver(s) during a phone call, video call, and/or in-person appointment.
- I expect the caregiver(s) family and/or friends to translate the recommendations for them.
- I request a formally translated version of the evaluation report to be sent to the parent.
- I provide an oral summary of the evaluation recommendations for the caregiver(s) during a phone call, video call, and/or in-person appointment.
- I do not know
- I prefer not to answer
- Other (describe below)

How do you currently communicate evaluation recommendations to caregivers who primarily speak/read Spanish? ________________________________________________________

To what degree would implementing the Spanish evaluation letter template be useful in your own clinical practice?

- Not useful at all
- Somewhat useful
- Completely useful

To what degree do you believe that there is a need to use the Spanish evaluation letter template in clinical practice?

- No need at all
- Some need
- Strong need

To what degree do you want to use the Spanish evaluation letter in your own clinical practice?

- I do not want to use it at all
- I somewhat want to use it
- I definitely want to use it

Do you evaluate patients whose caregivers primarily speak/understand Spanish, either as a Qualified Bilingual Staff member or with the help of an interpreter?

- Yes
- No

What do you suspect to be the benefit(s) of such a template? ___________________________

What do you suspect to be the drawback(s) of such a template? _________________________

In the next four (4) months, do you expect to evaluate patients whose caregivers primarily speak/understand Spanish, either as a Qualified Bilingual Staff member or with the help of an interpreter?

- Yes
- No

Are you willing to implement the Spanish evaluation letter in your clinical practice over the next four (4) months?

- Yes*
- No**

*You selected "yes" to the questions above (Do you [expect to] evaluate patients of caregivers who primarily speak/understand Spanish...; Are you willing to implement the Spanish evaluation letter...). As a result, you will receive additional information regarding this study and a post-survey to complete at the end of the four (4) month study to provide your feedback on the feasibility, acceptability, and appropriateness of this tool.

**You selected "no" to the questions above (Do you [expect to] evaluate patients of caregivers who primarily speak/understand Spanish...; Are you willing to implement the Spanish evaluation letter...). As a result, you will receive additional information regarding the Spanish evaluation letter as well as a general survey to provide your feedback regarding the feasibility, acceptability, and appropriateness of this tool.

**Second Clinician Survey**

Did you use the Spanish evaluation letter with your own patients’ caregivers over the course of this study? You will be asked a series of questions regardless of your answer to this question.

- Yes
- No

Are you a Qualified Bilingual Staff Member at [hospital name]?

- Yes
- No
- Prefer not to answer

If you are a Qualified Bilingual Staff Member, in what language are you qualified to interact with patients?

- Spanish
- Portuguese
- Mandarin
- Russian
- Other

If you selected Other, what language are you qualified to use with patients at [hospital name]? _____________________________________________________________________________

Do you use interpreter services (e.g., [interpretation vendor], staff interpreter] to review evaluation results and recommendations with your patients’ caregivers?

- Yes
- No
- Prefer not to answer

As best you can, please select the languages for which you solicit the assistance of interpreter services.

- Spanish
- Portuguese
- Russian
- Khmer
- Arabic
- Mandarin
- Other

If you selected “Other” for the previous question, please write below the languages for which you solicit the assistance of interpreter services (e.g., [interpretation vendor], staff interpreter) to the best of your ability. __________________________________________________

*Acceptability*

To what degree does the translated Spanish evaluation letter meet your approval?

- Does not meet my approval
- Somewhat meets my approval
- Completely meets my approval

To what degree is the translated Spanish evaluation letter appealing to you?

- Not at all appealing
- Somewhat appealing
- Completely appealing

To what degree do you like the translated Spanish evaluation letter?

- I do not like it at all.
- I somewhat like it.
- I like it very much.

To what degree do you welcome the translated Spanish evaluation letter?

- I do not welcome it at all.
- I somewhat welcome it.
- I welcome it very much.

What did you like about the translated Spanish evaluation letter? If you prefer not to respond, please write “prefer not to respond.” __________________________________________

What didn’t you like about the translated Spanish evaluation letter? If you prefer not to respond, please write “prefer not to respond.” __________________________________________

*Intervention Appropriateness*

To what degree does the translated Spanish evaluation letter seem fitting?

- Not fitting at all
- Somewhat fitting
- Completely fitting

To what degree does the translated Spanish evaluation letter seem suitable?

- Not suitable at all
- Somewhat suitable
- Completely suitable

To what degree does the translated Spanish evaluation letter seem applicable?

- Not applicable at all
- Somewhat applicable
- Completely applicable

To what degree does the translated Spanish evaluation letter seem like a good match?

- Not a good match at all
- Somewhat of a good match
- Completely good match

*Feasibility*

To what degree does the translated Spanish evaluation letter seem implementable?

- Not implementable at all
- Somewhat implementable
- Completely implementable

To what degree does the translated Spanish evaluation letter seem possible?

- Not possible at all
- Somewhat possible
- Completely possible

To what degree does the translated Spanish evaluation letter seem doable?

- Not doable at all
- Somewhat doable
- Completely doable

To what degree does the translated Spanish evaluation letter seem easy to use?

- Not easy to use at all
- Somewhat easy to use
- Completely easy to use

What are aspect(s) of the translated evaluation letter that made it easy to use? If none, please write “N/A.” If you prefer not to respond, please write “prefer not to respond.” ______________________________________________________________________________

What are aspect(s) of the translated evaluation letter that made it difficult to use? If none, please write “N/A.” If you prefer not to respond, please write “prefer not to respond.”

______________________________________________________________________________

*Benefits, Barriers, Follow Up Questions*

In the response box below, please provide any suspected barriers to implementing such a tool in your clinical practice. __________________________________________________________

In the response box below, please provide any suspected benefits to implementing such a tool in your clinical practice. __________________________________________________________

In the response box below, please provide any suggested adaptations to the tool.

______________________________________________________________________________

In the response box below, please provide any follow-up questions you have about this tool. ______________________________________________________________________________

**Caregiver Survey (Spanish Version)**

¿Cuál es su idioma dominante (o sea, el idioma del que tiene más dominio y/o el que usa con más frecuencia)?

- Ingles
- Español
- Portugués
- Otro

Si seleccionó “Otro,” especifique aquí su idioma dominante _____________________________

¿Es su idioma preferido (o sea, el idioma mayormente preferido para comunicarse) diferente a su idioma dominante?

- Sí
- No
- Prefiero no responder

¿Cuál es su idioma preferido (o sea, el idioma mayormente preferido para comunicarse)?

- Ingles
- Español
- Portugués
- Otro

Si seleccionó “Otro,” especifique aquí su idioma preferido _______________________________

¿Qué edad tiene su niño/a que fue evaluado por el Departamento del habla de [hospital name]?

- Menor de 2 años
- Entre 2 y 3 años
- Entre 3 y 5 años
- Mayor de 5 años

*Comentarios acerca de la carta de evaluación*

¿Recibió una carta en español con los resultados de la evaluación del habla y language de su niño/a?

- Sí
- No
- No estoy seguro/a
- Prefiero no responder

¿Qué tan útil le resultó recibir los resultados de la evaluación de su niño/a en español?

- Para nada útil
- Un poco útil
- Muy útil
- Prefiero no responder

¿Qué tan fácil fue entender los resultados en español?

- Para nada fácil
- Un poco fácil
- Muy fácil
- Prefiero no responder

¿Qué tan probable es que usted recomiende a otra familia que soliciten esta carta para los resultados de la evaluación de su propio niño/a?

- Improbable
- Un poco probable
- Muy probable
- Prefiero no responder

¿Qué tanto beneficio le brindó recibir estos resultados como parte del proceso de evaluación de su niño/a?

- Ningún beneficio
- Un poco de beneficio
- Gran beneficio
- Prefiero no responder

¿Qué tan fácil fue entender los resultados en español?

- Para nada difícil
- Un poco difícil
- Muy difícil
- Prefiero no responder

¿Qué tan satisfecho estuvo con la capacidad de esta carta para explicar los resultados de la evaluación?

- Para nada satisfecho
- Un poco satisfecho
- Muy satisfecho
- Prefiero no responder

En el cuadro a continuación, describa algún resultado de la evaluación que usted considere que no se reflejó en la carta. __________________________________________________________

¿Qué tan satisfecho estuvo con la capacidad de esta carta de explicar las recomendaciones para su niño/a?

- Para nada satisfecho
- Un poco satisfecho
- Muy satisfecho
- Prefiero no responder

En el cuadro a continuación, describa alguna recomendación de la evaluación que usted considere que no se reflejó en la carta. ______________________________________________

¿Cuál cree que podría ser un impacto positivo de que las familias tengan acceso a esta carta?

______________________________________________________________________________

¿Cuál cree que podría ser un impacto negativo de que las familias tengan acceso a esta carta?

______________________________________________________________________________

¿Cuál es al menos una forma en la que se puede mejorar esta carta?

______________________________________________________________________________

Comparta aquí cualquier duda o pregunta que tenga sobre la carta.

______________________________________________________________________________

**Caregiver Survey (English Translation)**

*Demographic Information*

Please answer these questions about yourself (caregiver).

What is your dominant language (i.e., the language with you have greatest proficiency and/or use most often)?

- English
- Spanish
- Portuguese
- Other

If selected “Other,” please specify your dominant language here. ________________________

Is your preferred language (i.e., language most preferred for communication) different than your dominant language?

- Yes
- No
- Prefer not to answer

What is your preferred language (i.e., the language most preferred for communication?

- English
- Spanish
- Portuguese
- Other

If you selected “Other,” please specify your preferred language below. ___________________

How old is your child who was evaluated by the [hospital name] Speech Department?

- Younger than 2 years old
- Between 2 and 3 years old
- Between 3 and 5 years old
- Older than 5 years old

*Feedback about Evaluation Letter*

Did you receive a letter in Spanish with your child’s speech-language evaluation results?

- Yes
- No
- Not sure
- Prefer not to answer

How helpful was it to receive your child’s evaluation results in Spanish?

- Not helpful at all
- Somewhat helpful
- Very helpful
- Prefer not to answer

How easy were the evaluation results to understand in Spanish?

- Not easy at all
- Somewhat easy
- Very easy
- Prefer not to answer

How likely are you to recommend to another family that they request this letter for their own child’s evaluation results?

- Not likely
- Somewhat likely
- Very likely
- Prefer not to answer

How much of a benefit did receiving these results provide as a part of your child’s evaluation process?

- No benefit at all
- Somewhat of a benefit
- Significant benefit
- Prefer not to answer

How difficult were the evaluation results to understand in Spanish?

- Not difficult at all
- Somewhat difficult
- Very difficult
- Prefer not to answer

How satisfied were you with the ability of this letter to explain the results of the evaluation?

- Not satisfied at all
- Somewhat satisfied
- Very satisfied
- Prefer not to answer

In the box below, please describe any of the evaluation results that you felt were not reflected in the letter. ________________________________________________________________

How satisfied were you with the ability of this letter to explain the recommendations for your child?

- Not satisfied at all
- Somewhat satisfied
- Very satisfied
- Prefer not to answer

In the box below, please describe any of the evaluation recommendations that you felt were not reflected in the letter. _______________________________________________________

What do you think could be a positive impact of families having access to this letter?

______________________________________________________________________________

What is at least one way in which this letter can be improved?

______________________________________________________________________________

Please share any of the questions you have about this letter.

______________________________________________________________________________
